# Author Correction: Representation of molecular structures with persistent homology for machine learning applications in chemistry

**DOI:** 10.1038/s41467-020-17423-x

**Published:** 2020-07-14

**Authors:** Jacob Townsend, Cassie Putman Micucci, John H. Hymel, Vasileios Maroulas, Konstantinos D. Vogiatzis

**Affiliations:** 1 Department of Chemistry, University of Tennessee, Knoxville, TN 37996-1600 USA; 2 Department of Mathematics, University of Tennessee, Knoxville, TN 37996-1320 USA

**Keywords:** Cheminformatics, Computational chemistry, Applied mathematics

Correction to: *Nature Communications* 10.1038/s41467-020-17035-5, published online 26 June 2020.

 In the original version of this article, some text was missing from the legends of Figure 5 and Figure 6.

The legend of Figure 5 originally read:

“CO_2_ interaction energy distribution shown as horizontal violin plots for the first, second, and third active-learning steps. The height of the shape shows the frequency of occurrences..”

The correct version states:

“CO_2_ interaction energy distribution shown as horizontal violin plots for the first, second, and third active-learning steps. **a** CM, **b** BoB, **c** SOAP, and **d** PI. The height of the shape shows the frequency of occurrences…”

This has been corrected in both the PDF and HTML version of the article.

The legend of Figure 6 originally read**:**

“Predicted CO_2_ and N_2_ interaction energies (in kcal mol^−1^) for all molecules in the GDB-9 database using four molecular representation models. Only the model that utilized the PI molecular representation …”

The correct version states:

“Predicted CO_2_ and N_2_ interaction energies (in kcal mol^−1^) for all molecules in the GDB-9 database using four molecular representation models. **a** CM, **b** BoB, **c** SOAP, and **d** PI. Only the model that utilized the PI molecular representation.”

This has been corrected in both the PDF and HTML version of the article.

