# Supplementary material for: Representation of molecular structures with persistent homology for machine learning applications in chemistry
Source: Nat Commun. 2020 Jun 26;11:3230. doi: 10.1038/s41467-020-17035-5 (PMC7319956; doi:10.1038/s41467-020-17035-5)
Supplement: Supplementary file 1 — Supplementary Information [file 41467_2020_17035_MOESM1_ESM.pdf]

SUPPLEMENTARY INFORMATION

Representation of Molecular Structures with Persistent Homology for Machine Learning Applications in Chemistry

Townsend *et al.*

Supplementary Note 1. Application of Persistent Homology on Molecular Systems

For each molecule, persistence diagrams associated with connected components and holes are computed using the Vietoris-Rips complex using the Ripser python package.[13] The persistence diagrams use *(birth, persistence)* coordinates of the respective atoms, where *persistence = death - birth* represents the length of the lifetime (*persistence*) of a homological feature.

Once the persistent diagrams have been constructed, we generate the persistent images (PIs) by considering for each point in the diagram the additive Gaussian *(birth, persistence)*$+N(0, \sigma^2 \cdot SF_{ij})$, where N is the normal distribution. For points on the diagram associated to connected components, this variance is scaled by the difference in electronegativity between the atoms i and j, given by the following formula:

$$SF_{ij} = \frac{|EN_i - EN_j| + \varepsilon}{10}, \quad (1)$$

where EN_i and EN_j represent the electronegativity of atoms i and j. The parameter ε of the scaling factor SF_{ij} prevents the variance from becoming zero when atoms i and j are equivalent. A value of $\varepsilon = 0.4$ was found to be the optimal. These scaling factors modify the default Gaussian variance, for which we found that $\sigma^2 = 0.08$ to perform best for this application. For holes, no scaling based on electronegativity was necessary ($SF_{ij} = 1$), and a variance of $\sigma^2 = 0.08$ was used.

The points of each persistence diagram along with the variances assigned to each point, are then input in a modified version of the Persim python package.[14] The code is changed to use fixed boundaries for the persistence vectors and images. Persistence vectors are then calculated over the interval from -0.1 to the 2.5 of the persistence values. The variance corresponding to each point is then used as the variance for the Gaussian centered at that point for the persistence vector or image. Persistence vectors are discretized into 150 horizontal and vertical pixels to form the persistence images, corresponding to vector length of 22500. No weighting function was used in the calculations of the persistence vectors or persistence images.

Supplementary Note 2. Similar-size Representation of Persistent Images

A numerical example is given that describes the similar-size molecular representation that the persistence images (PI) offer and compared to the bag-of-bonds (BoB) method. For demonstrating this feature of the PI, which is a smeared version of a persistence diagram (PD), we compare the dimensions of these two representations for a small molecule (anisole), a medium-size molecule (4-*tert*-butylcalix[4]arene, Supplementary Figure *1*), and a large enzyme (main protease in complex with an inhibitor N3 of COVID-19). Anisole is composed by 16 atoms, thus, the Coulomb matrix that is constructed for BoB is of 16x16 size. 4-*tert*-butylcalixarene has 104 atoms, thus the same matrix will be of 104x104 dimension. On the contrary, a PD for anisole has 3x3 dimension (Figure 1 from main text), while a PD for a calixarene slightly increases to a 4x4 representation (Supplementary Figure *1* (c), highest birth value is 3.93). Thus, the amount of padding (i.e. adding zeros) is significantly smaller. Similarly, the PD of the main protease in complex with an inhibitor N3 of the new coronavirus (CoV) identified as COVID-19 is shown in Supplementary Figure *2*. The crystal structure of the complex was used.[15] For simplicity, the hydrogen atoms were omitted, leading to a biomolecule with 2500 atoms (2367 atoms from protease, 49 from inhibitor, and 84 oxygen atoms of water molecules). The Coulomb matrix will have 2500x2500 size, while the PD has a significantly smaller dimension (4Åx6Å), which is comparable with the PDs of anisole and calixarene.

Note: Our code, representative examples, and all structures in xyz format can be conveniently downloaded at https://gitlab.com/voglab/PersistentImages_Chemistry

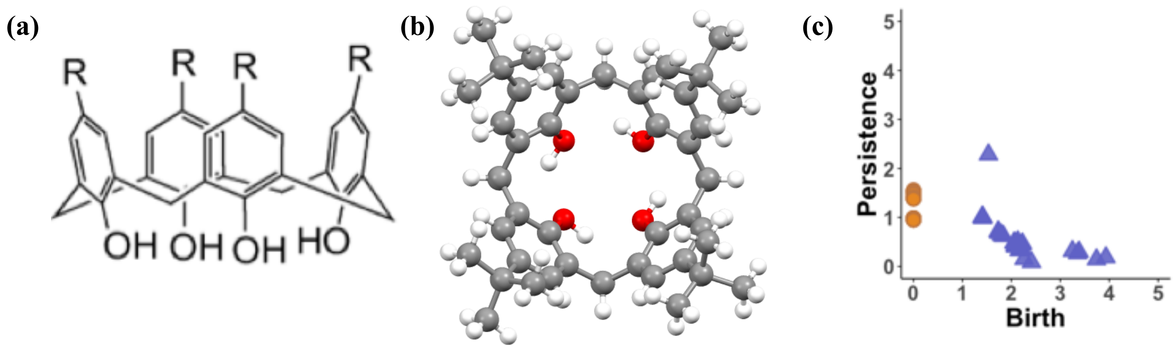

Supplementary Figure 1. (a) Structural formula and (b) ball-and-stick model of 4-tert-butylcalix[4]arene (R = tert-butyl groups). (c) The persistent diagram of the 4-tert-butylcalix[4]arene. Note that the highest birth value of a one-dimensional hole is 3.93.

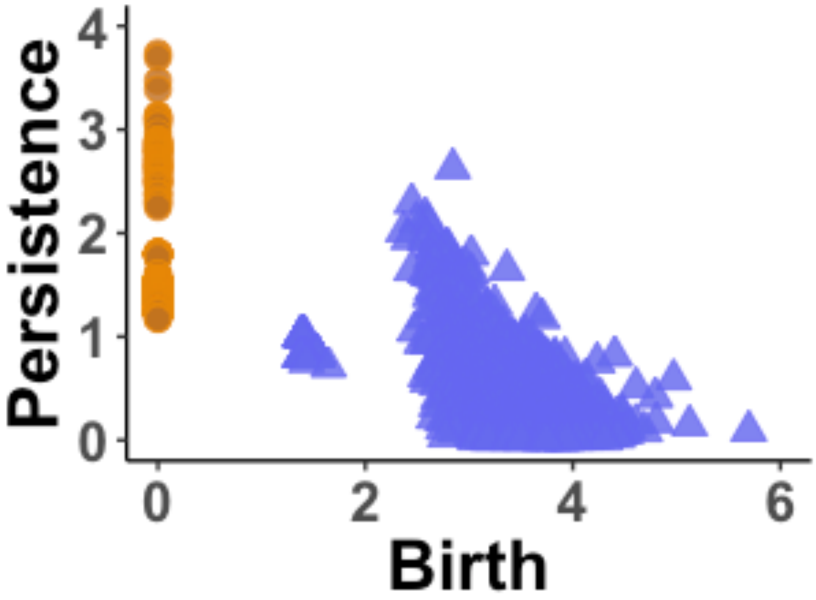

Supplementary Figure 2: The persistent diagram of the COVID-19 main protease in complex with an inhibitor N3 (hydrogen atoms are omitted).

Supplementary Note 3. Molecular Subset Selection

To demonstrate the applicability of the novel molecular fingerprinting method, a dataset containing 100 organic aromatic molecules was compiled. All molecules considered here are composed of C, O, N, F, S and H atoms. The interaction energies of each molecule with CO_2 and N_2 were computed by combining molecular dynamics simulations and density functional theory (DFT) for the screening of different conformers and interaction sites[1], and are summarized in Supplementary Notes 4 and 5.

Supplementary Note 4. Molecular Dynamics Conformation Search

The conformational prescreening step offers an automated conformation search between competitive local minima of the full potential energy surface. An example of competitive binding sites is given for 7-methyl-imidazopyridine (imidazopyridamine, structure 10 of the 100-molecule dataset) in Supplementary Figure 3. Prescreening of the conformational space with molecular dynamics simulations provided many unbiased initial geometries which were further optimized by DFT. Although interaction energies are calculated at the DFT level, which entails a thorough conformational search to determine the most favorable binding site of a respective gas with the structure, only the structure in the absence of the interacting gas is passed to the molecular representation. Therefore, the ML algorithm will infer the interaction energy of the most favorable binding site without any explicit knowledge of the gas, therefore bypassing the need of intensive conformational searches at the time of prediction. These structures were generated in a 16 Angstrom box at 200 K in the NVT ensemble with a Nose-Hoover thermostat using a 1.0 ps time-constant and a 1.0 fs time-step. The OPLS-AA force field[2] was used, where LigParGen[3] was applied for the generation of force-field input implemented in the LAMMPS software package[4] Thirty structures were considered for each functional group (monomer) and monomer-gas supersystems, resulting in a total 3*30*100 = 9000 DFT geometry optimizations.

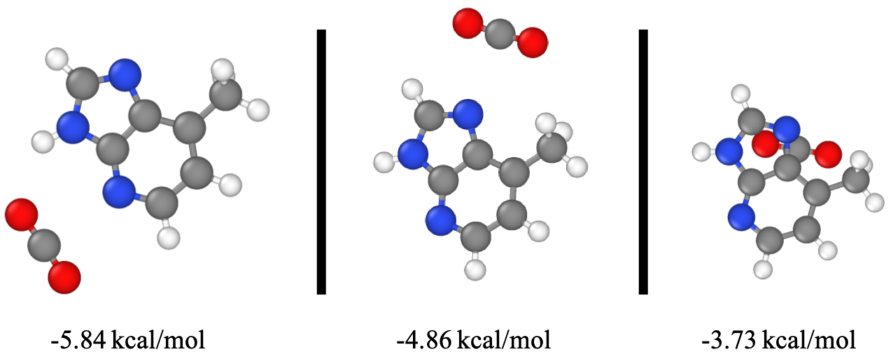

Supplementary Figure 3. Three different conformations of 7-methyl-imidazopyridine with CO_2. Each binding site has considerably different interaction energies, which demonstrates the importance of sufficient sampling of conformations.

Supplementary Note 5. Density Functional Theory Calculations

All DFT calculations were performed with the TURBOMOLE 7.2 program package[5] using the PBE0 functional[6] with the def2-TZVPP basis set[7]. Grimme's D3 dispersion correction[8] with the Becke-Johnson damping function[9] was included to account for dispersion effects. The choice of the PBE0-D3(BJ)/def2-TZVPP level of theory was chosen based on benchmark studies between functional groups and CO_2.[10,11] Integral evaluations were performed with an ultrafine grid, where the resolution of identity was utilized in the computations of two-electron integrals.[12] All DFT geometry optimizations were performed with tight convergence criteria, and frequency calculations were performed on structures to ensure they are minima on the potential energy surface. Interaction energies ΔE_{Int} between a gas molecule with a functional group were calculated as:

$$\Delta E_{Int} = E_{Organic-Gas} - E_{Organic} - E_{Gas}$$

where $E_{Organic-Gas}$ represents the energy of the organic-gas molecular supersystem, while $E_{Organic}$ and E_{Gas} represent the energies of the relaxed isolated organic and gas molecules, respectively.

Supplementary Note 6. Testing of Different Molecular Representations and Machine Learning Methods

The SciKitLearn[16] package was used for the machine learning. The following acronyms will be used for the learners:

Supplementary Table 1. Acronyms for learners corresponding to the SciKitLearn package

RF	RandomForestRegressor
GPR	GaussianProcessRegressor (Matern 5/2 kernel)
KRR linear	KernelRidge(kernel='linear')
KRR RBF	KernelRidge(kernel='rbf')
KRR Laplacian	KernelRidge(kernel='laplacian')

Smooth Overlap of Atomic Positions[17] representations were created using the dscribe[18] program package. SOAP feature vectors are atomistic, therefore a transformation is necessary for molecular analyses. The REMatch kernel was used to find similarity of local environments of molecules as implemented in dscribe.[19] An 8.0 Å cutoff was used in the generations of the SOAP representation, with 4 radial basis functions (nmax=4) and a maximum degree of spherical harmonics of 4 (lmax=4). The REMatch Kernel was implemented with a Gaussian kernel with default hyperparameters (gamma=1, alpha=1).

The BoB[20], CM[21], and FCHL[22] representations were generated in QML.[23] For the FCHL representation, kernel ridge regression within QML was used for prediction. The python package Ripser[13] was used for the generation of persistence diagrams (PDs). The persistence images were generated with a modified version of the persim python package.[14] Catenated persistence images (PIs) were created with 150 horizontal and vertical pixels, corresponding to feature vectors of length 22,500. 0 and 1-dim features on the PD were placed on a single persistence image, ranging from -0.1 to 2.5 Å along the image. The negative x-limit was included to allow the 0-dim gaussians to fully extend without truncation.

Timings are reported in seconds of calculation time to predict the interaction energies of the entire GDB-9 database on an i5-4278U processor. Times are reported for CO_2 interactions only, as the timings are equivalent because the representations are unchanged.

Supplementary Table 2. 10-fold cross validation RMSE and standard deviations, timings, and optimized hyperparameters for Coulomb Matrix (CM) Representation for the prediction of CO_2 Interaction Energies

CM	RF	GPR	KRR linear	KRR RBF	KRR Laplacian
Average RMSE (kcal/mol)	0.67	0.64	0.76	0.73	**0.63**
SD	0.13	0.10	0.10	0.14	0.09
Alpha	N/A	2.15E-01	3.24E+00	1.21E-01	3.59E-02
Gamma	N/A	N/A	N/A	2.22E-03	4.64E-03
Time (s)	190.56	196.54	125.72	165.73	150.46

Supplementary Table 3. 10-fold cross validation RMSE and standard deviations, timings, and optimized hyperparameters for Bag of Bonds Representation for the prediction of CO_2 Interaction Energies

BoB	RF	GPR	KRR linear	KRR RBF	KRR Laplacian
Average RMSE (kcal/mol)	0.54	**0.52**	0.58	0.67	0.53
SD	0.15	0.10	0.10	0.27	0.09
Alpha	N/A	1.00E-01	4.29E+00	4.18E-03	4.94E-03
Gamma	N/A	N/A	N/A	2.15E-03	5.18E-04
Time (s)	122	**191**	91	97	120

Supplementary Table 4. 10-fold cross validation RMSE and standard deviations, timings, and optimized hyperparameters for the Persistence Images for the prediction of CO_2 Interaction Energies

PI	RF	GPR	KRR linear	KRR RBF	KRR Laplacian
Average RMSE (kcal/mol)	0.49	0.51	0.63	0.50	**0.44**
SD	0.12	0.13	0.15	0.10	0.09
Alpha	N/A	4.64E-02	1.67E-01	5.99E-03	2.15E-07
Gamma	N/A	N/A	N/A	4.64E-02	3.59E-02
Time (s)	1571	2091	1595	1515	**2219**

Supplementary Table 5. 10-fold cross validation RMSE and standard deviations, timings, and optimized hyperparameters for the FCHL representation for the prediction of CO_2 Interaction Energies

FCHL	KRR RBF
Average RMSE (kcal/mol)	**0.50**
SD	0.10
Sigma	1750
Lambda	1.00E-08
Time (s)	**46782**

Supplementary Table 6. 10-fold cross validation RMSE and standard deviations, Timings, and optimized hyperparameters for the SOAP representation for the prediction of CO_2 Interaction Energies

SOAP	RF	GPR	KRR linear	KRR RBF	KRR Laplacian
Average RMSE (kcal/mol)	0.69	0.67	**0.41**	0.50	0.68
SD	0.12	0.13	0.11	0.08	0.13
Alpha	N/A	7.74E-02	6.55E-06	5.99E-05	6.83E-01
Gamma	N/A	N/A	N/A	4.64E-03	3.59E-03
Time (s)	94981	94745	**88287**	89263	92295

Supplementary Table 7. 10-fold cross validation RMSE and standard deviations and optimized hyperparameters for Coulomb Matrix Representation for the prediction of N_2 Interaction Energies

CM	RF	GPR	KRR linear	KRR RBF	KRR Laplacian
Average RMSE (kcal/mol)	0.23	**0.22**	0.27	0.29	0.24
SD	0.06	0.06	0.09	0.15	0.07
Alpha	N/A	2.15E-02	1.84E+00	7.20E-03	2.12E-03
Gamma	N/A	N/A	N/A	1.46E-01	2.22E-04

Supplementary Table 8. 10-fold cross validation RMSE and standard deviations and optimized hyperparameters for Bag of Bonds Representation for the prediction of N_2 Interaction Energies

BoB	RF	GPR	KRR linear	KRR RBF	KRR Laplacian
Average RMSE (kcal/mol)	0.23	**0.22**	0.27	0.27	0.24
SD	0.06	0.06	0.09	0.09	0.08
Alpha	N/A	2.15E-02	2.02E+00	4.64E-04	3.59E-03
Gamma	N/A	N/A	N/A	5.99E-05	7.74E-04

Supplementary Table 9. 10-fold cross validation RMSE and standard deviations and optimized hyperparameters for Persistence Image Representation for the prediction of N_2 Interaction Energies

PI	RF	GPR	KRR linear	KRR RBF	KRR Laplacian
Average RMSE (kcal/mol)	0.22	0.23	0.33	0.23	**0.22**
SD	0.06	0.07	0.09	0.08	0.06
Alpha	N/A	7.74E-03	2.15E-01	1.00E-02	1.00E-04
Gamma	N/A	N/A	N/A	4.64E-02	4.64E-02

Supplementary Table 10. 10-fold cross validation RMSE and standard deviations optimized hyperparameters for the FCHL representation for the prediction of N_2 Interaction Energies

FCHL	KRR RBF
Average RMSE (kcal/mol)	**0.22**
SD	0.04
Sigma	2000
Lambda	1.00E-08

Supplementary Table 11. 10-fold cross validation RMSE and standard deviations and optimized hyperparameters for the SOAP Representation for the prediction of N_2 Interaction Energies

SOAP	RF	GPR	KRR linear	KRR RBF	KRR Laplacian
Average RMSE (kcal/mol)	0.32	0.28	0.21	**0.20**	0.30
SD	0.07	0.07	0.05	0.04	0.07
Alpha	N/A	2.15E-02	2.12E-04	7.74E-08	5.99E-07
Gamma	N/A	N/A	N/A	7.74E-05	1.29E-02

Supplementary Note 7. N_2 Deviations from Different Molecular Representation Schemes

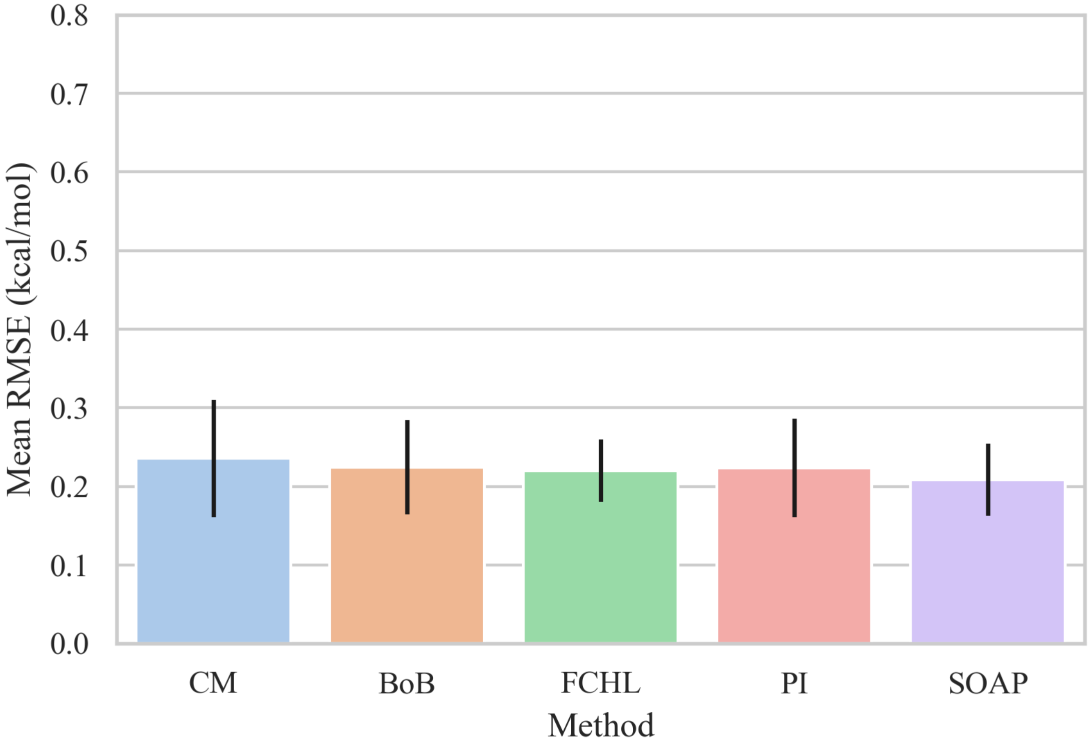

Supplementary Figure 4. Average RMSE (in kcal/mol) using 10-fold CV on N_2 interaction energies for Coulomb Matrices (CM), Bag of Bonds (BoB), FCHL representation, Persistence Images (PI), and Smooth Overlap of Atomic Positions (SOAP) representations. The error bars represent the standard deviation of the RMSE. Overall, all models show similar behavior due to the small range of output values since N_2 interaction energies range roughly between -1.6 and -2.6 kcal/mol.

Supplementary Note 8. Active Learning

The performance of each respective ML models was evaluated with 10-fold cross validation, which partitions the data into 10 sets, where each of the 10 sets is used as the test set once while the other 9 serve as training. This technique provides a metric for the performance of the model on unseen data. All of the data is passed into the test set once, and the mean root mean squared error (RMSE) of the predictions on the test set are used to evaluate model performance. To ensure the ML-model contains sufficient data to accurately interpolate between the DFT-studied set and the GDB-9 database, active learning was utilized, where the training set is expanded to capture missing information from the previous training data set. The dataset was dynamically expanded by including the top 40 molecules with respect to ML-predicted CO_2 interaction strength and further investigated by the MD/DFT scheme described in the computational details (Supplementary Notes 4 and 5). Therefore, active learning provides a systematic expansion of the training set tailored towards molecules with high CO_2 interaction strength. This process was performed four times, considering the four different molecular representations discussed in the manuscript (CM, BoB, SOAP, PI). The active learning steps for each representation were performed with the most accurate learner, as it was found in Supplementary Note 6, i.e. KRR (Laplacian) for CM and PI, GRP for BoB, and KRR (linear) for SOAP.

Supplementary Table 12: Mean, median and top cases (stronger CO_2 interaction energy than -6.0 kcal/mol) for the top-40 structures per active learning iteration for the four different molecular representations examined in this study.

Method		CM	BoB	SOAP	PI
1st Iteration	Mean	-4.92	-4.64	-5.78	-5.67
	Median	-4.74	-4.65	-5.82	-5.81
	Top	3	2	16	10
2nd Iteration	Mean	-4.91	-5.22	-5.84	-6.32
	Median	-4.76	-5.20	-5.83	-6.46
	Top	1	4	15	33
3rd Iteration	Mean	-4.84	-4.76	-5.88	-6.35
	Median	-4.89	-4.64	-6.01	-6.57
	Top	2	2	20	32
Overall	Mean	-4.89	-4.88	-5.83	**-6.11**
	Median	-4.77	-4.77	-5.85	**-6.32**
	Top	6	8	51	**75**

Predicted CO_2/N_2 interaction energies from the BoB with the GRP machine learning algorithm provided unphysical distributions, as it is shown on Supplementary Figure *5*(a). In order to examine if the source of error was the choice of the learner or the quality of the training data, we screened the GDB-9 database with the BoB representation, the GPR learner, and data obtained from the CM active learning steps. This scheme also provided erroneous results (Supplementary Figure *5*(b)), but more reasonable N_2 distribution. On the contrary, when we screened the same database with the BoB representation, the KRR (linear kernel) learner and the data obtained from the BoB active learning steps, the expected CO_2/N_2 distributions were recovered (Supplementary Figure *5*(c)). Thus, we conclude that an overfitting towards the training set was due to the GPR

learner, and slightly better distributions were obtained when more balanced data were used (active learning with KRR/CM).

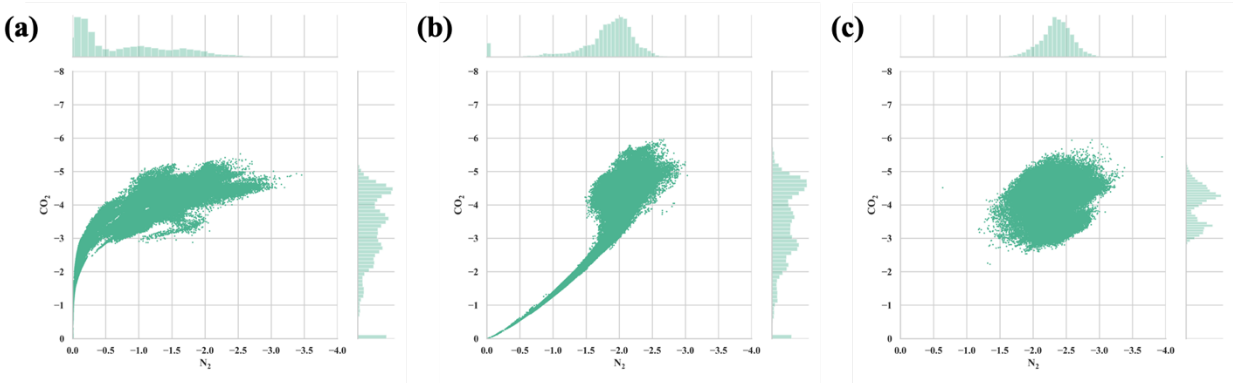

Supplementary Figure 5: Predicted CO_2 and N_2 interaction energies (in kcal/mol) for all molecules in the GDB-9 database using the BoB molecular representation and (a) the GRP learner with data from the BoB active learning steps, (b) the GRP learner with data from the CM active learning steps, and (c) the KRR (linear) with data from the BoB active learning steps.

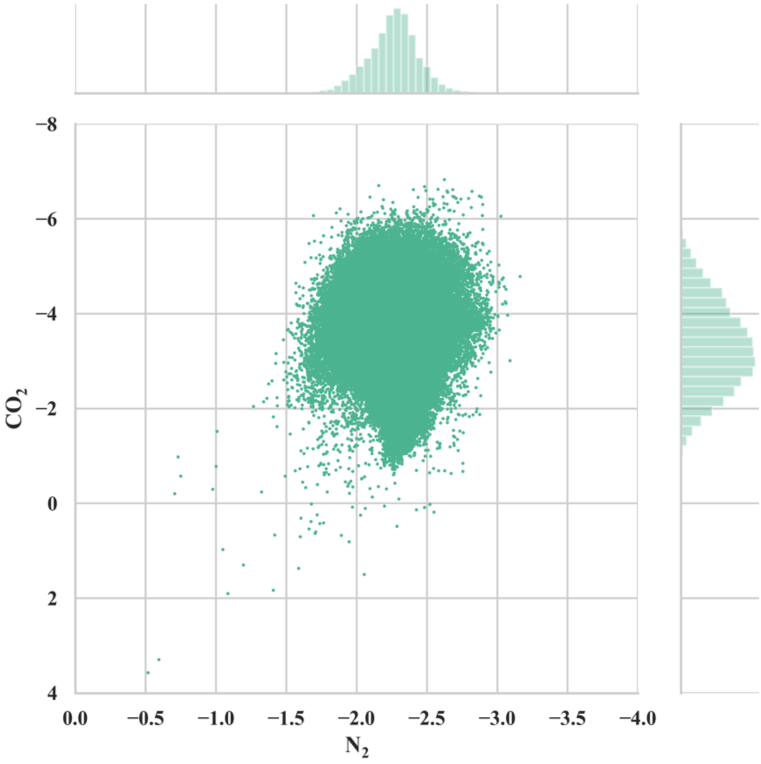

Supplementary Figure 6: Predicted CO_2 and N_2 interaction energies (in kcal/mol) for all molecules in the GDB-9 database using the SOAP molecular representation. Notice the erroneous predictions for organic molecules with repulsive interaction with CO_2 (positive interaction energies).

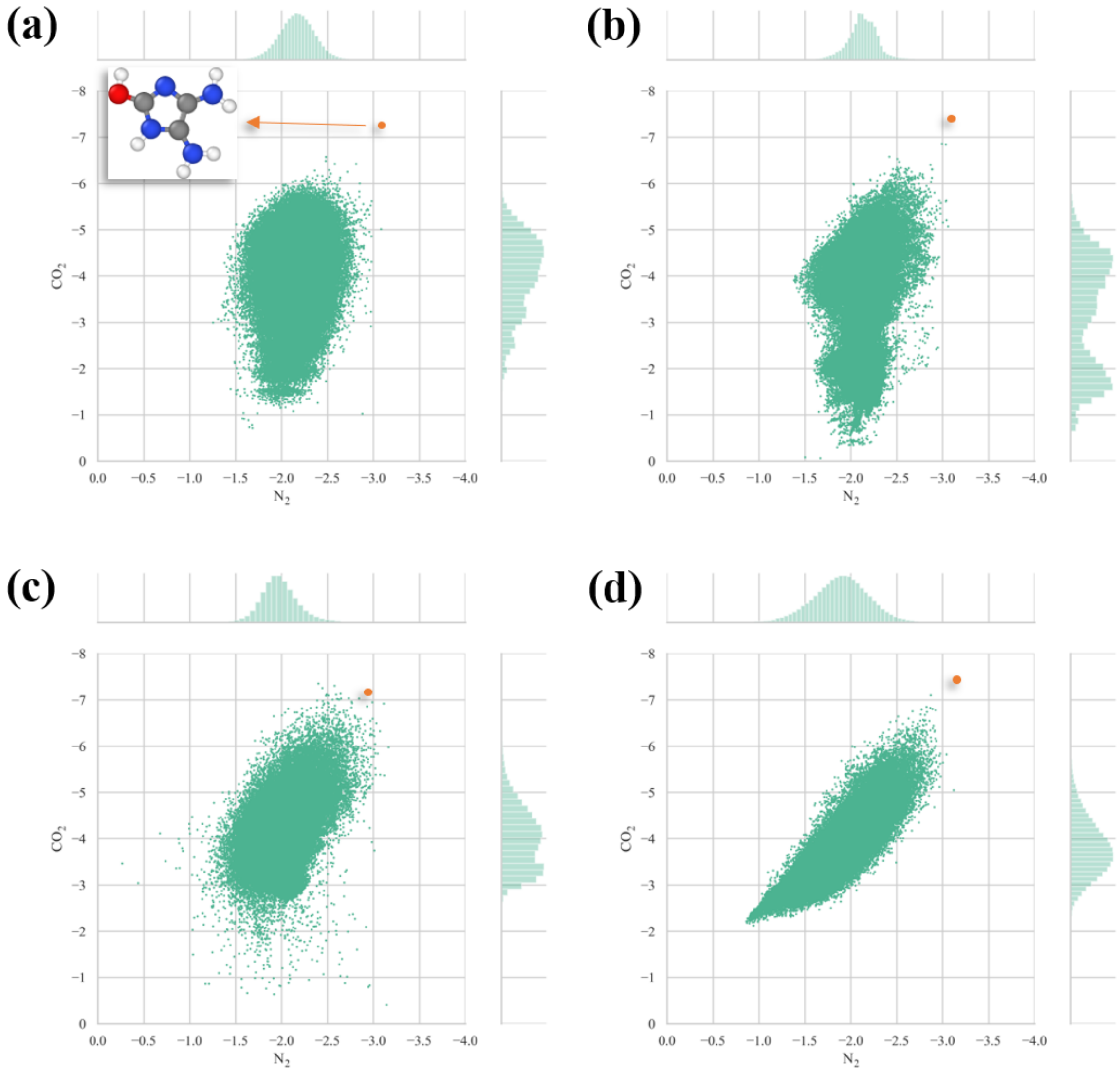

Supplementary Figure 7: Predicted CO_2 and N_2 interaction energies (in kcal/mol) for all molecules in the GDB-9 database using four molecular representation models: (a) CM, (b) BoB, (c) SOAP, and (d) PI. The training of each model was performed with data collected from the PI active learning steps and the same learner (random forest). All models were able to identify 4,5-diamino-1H-imidazol-2-ol (inset) as the strongest CO_2-philic groups (shown with an orange dot on each plot).

Supplementary Note 9. Interaction Energies and Structures for 100 Initial Molecules

Supplementary Table 13. 100 Initial Molecules used for the generation of ML models, their DFT interaction energies with CO_2 and N_2, the iteration of active learning they were introduced to the model, and a depiction of their chemical structure (made in RDKit, displayed formal charges may have inaccuracies)

Molecule #	CO_2 Interaction Energy	N_2 Interaction Energy	Structure
1	-4.58	-1.75	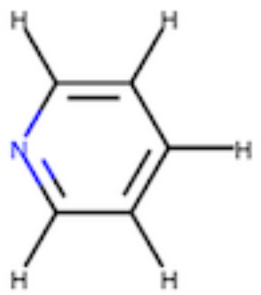
2	-4.12	-1.57	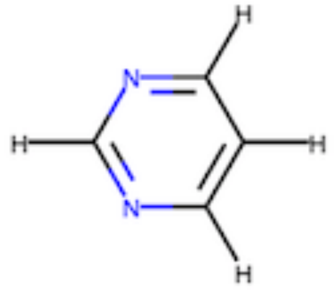
3	-4.02	-1.60	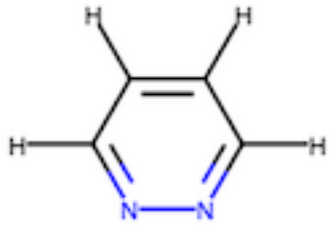

4	-4.08	-1.70	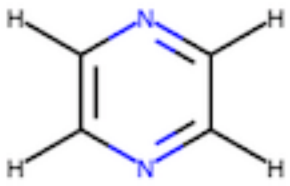
5	-3.65	-1.32	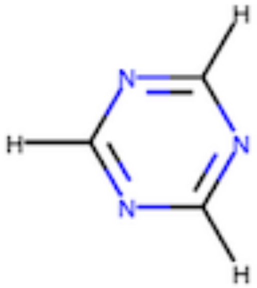
6	-4.57	-1.86	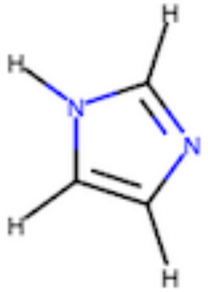
7	-3.99	-2.24	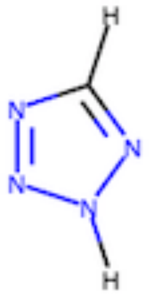

8	-5.49	-2.26	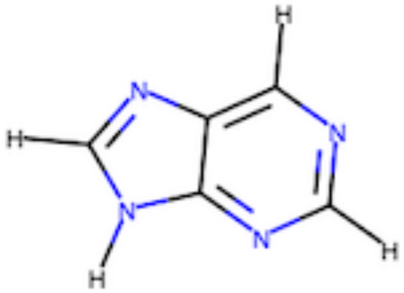
9	-5.73	-2.19	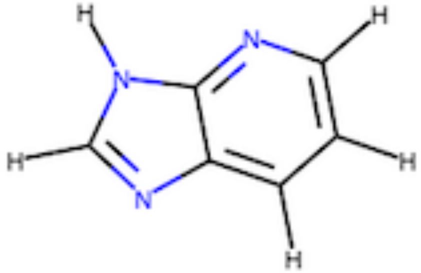
10	-5.84	-2.19	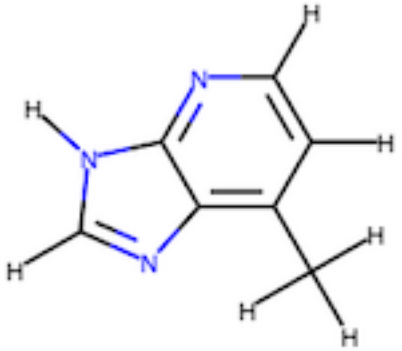
11	-5.60	-2.25	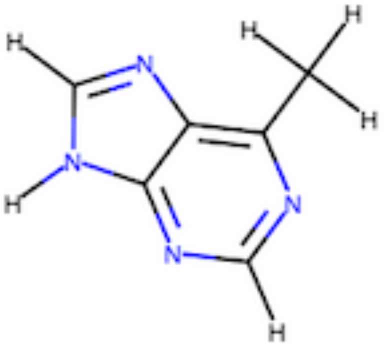

12	-2.64	-1.82	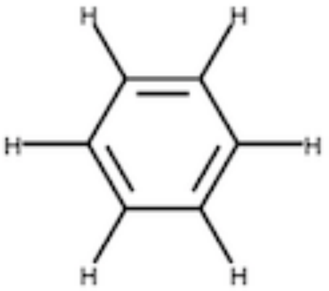
13	-4.75	-1.99	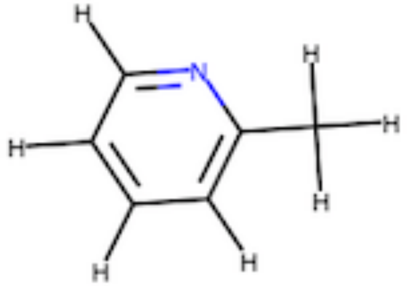
14	-4.34	-2.19	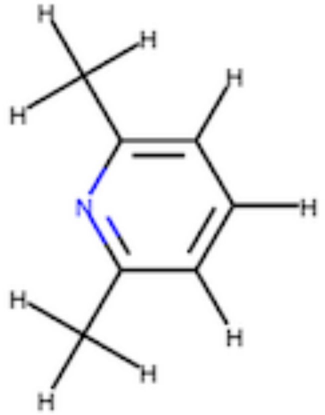

15	-4.43	-2.29	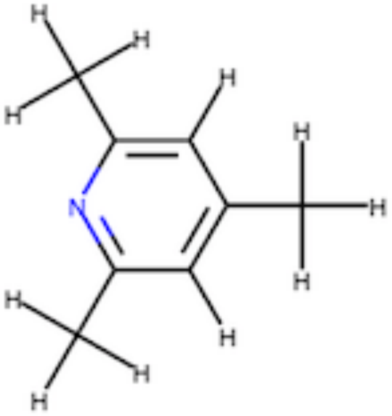
16	-4.74	-2.18	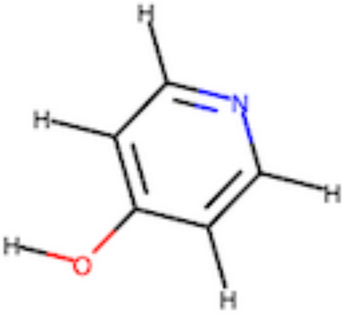
17	-5.64	-2.22	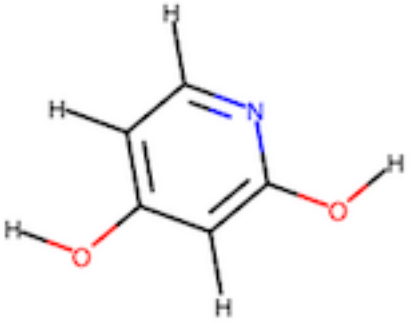
18	-6.07	-2.21	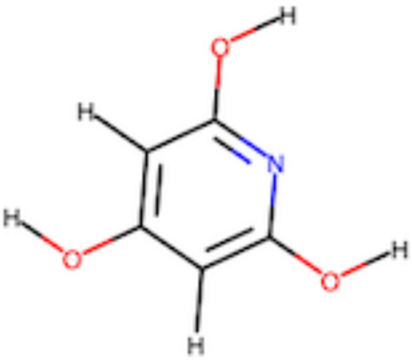

19	-4.79	-1.86	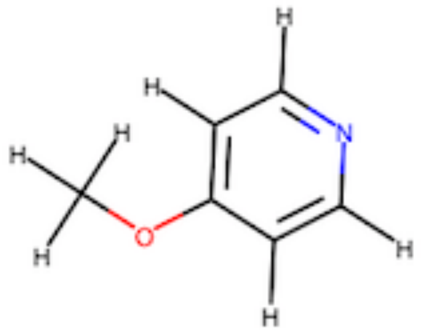
20	-3.79	-2.01	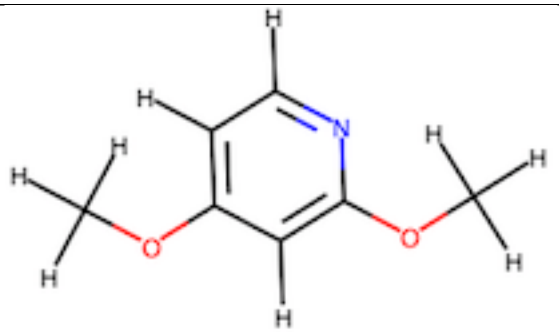
21	-3.74	-2.23	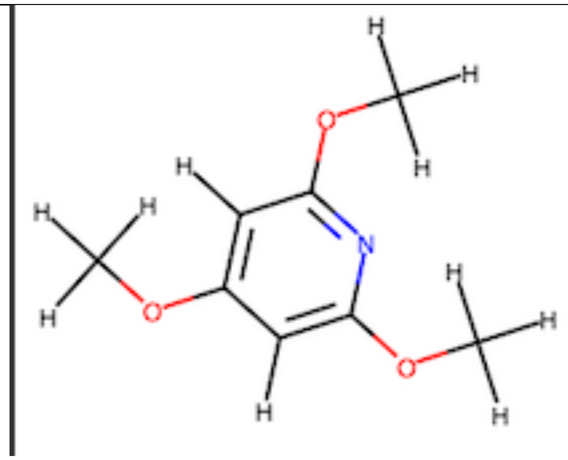
22	-5.68	-1.99	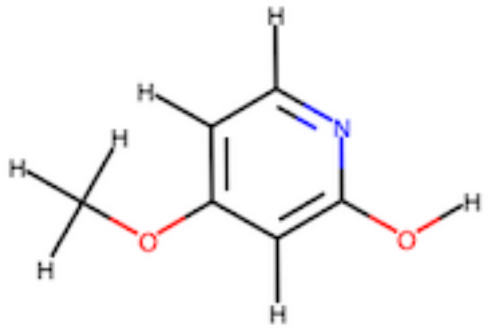

23	-6.08	-1.99	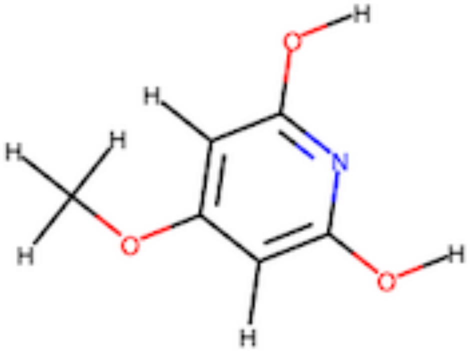
24	-4.40	-1.80	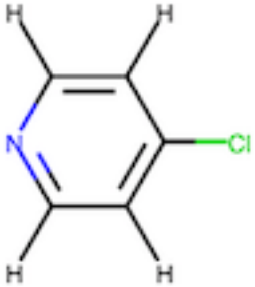
25	-4.47	-1.69	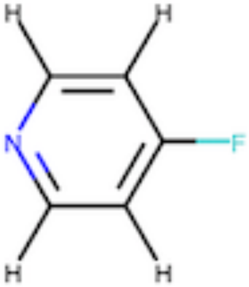
26	-4.23	-1.86	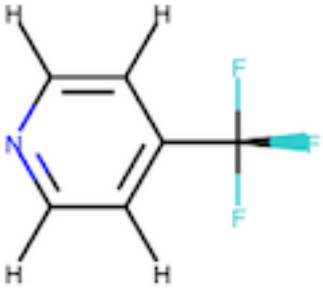

27	-3.77	-1.79	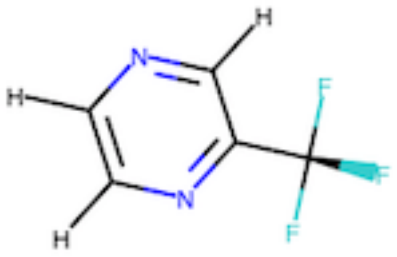
28	-5.23	-2.12	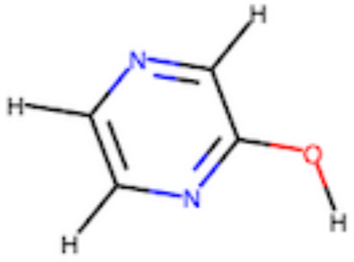
29	-6.91	-3.60	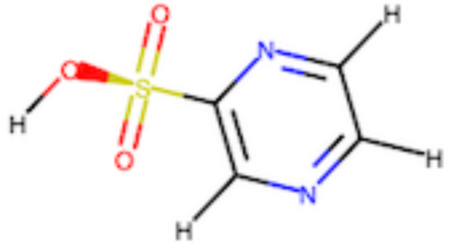
30	-4.37	-1.92	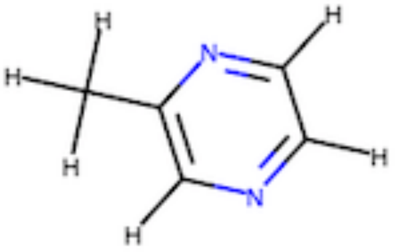

31	-3.96	-2.22	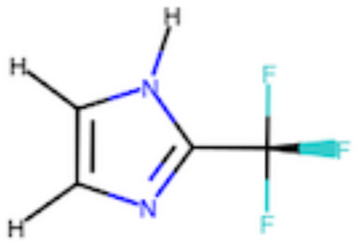
32	-4.77	-2.08	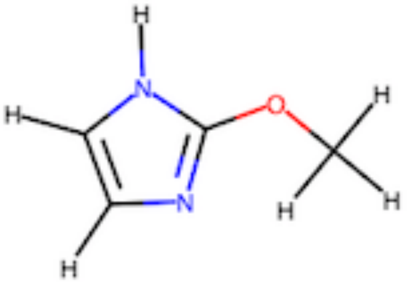
33	-4.63	-2.67	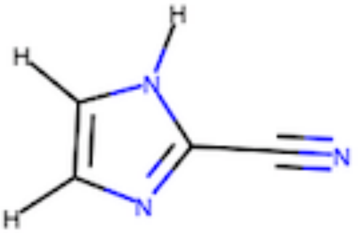
34	-7.28	-3.59	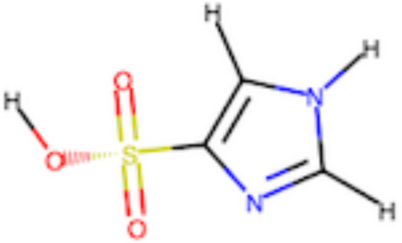

35	-3.83	-1.70	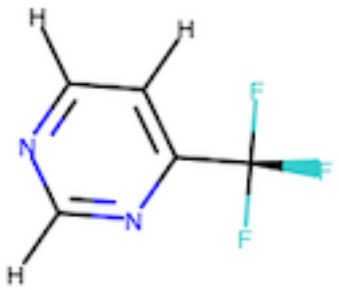
36	-4.35	-1.69	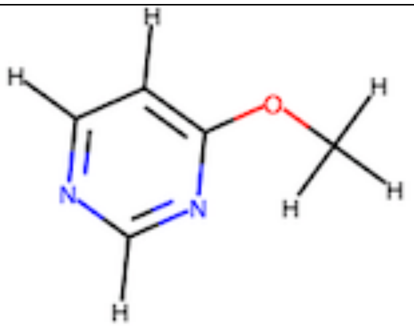
37	-3.73	-1.59	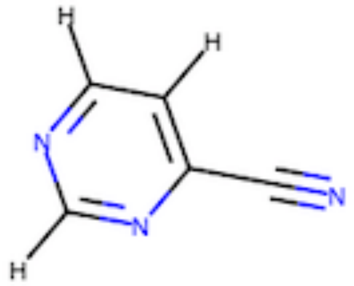
38	-6.88	-3.59	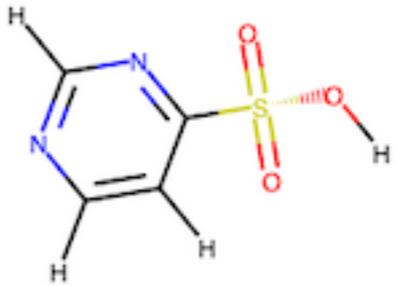
39	-5.33	-2.18	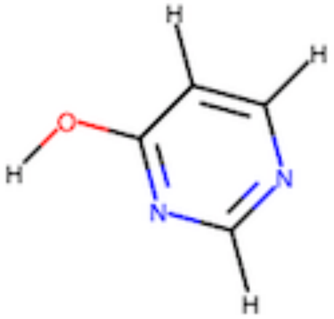

40	-3.74	-1.70	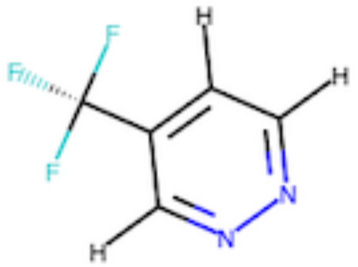
41	-4.15	-1.81	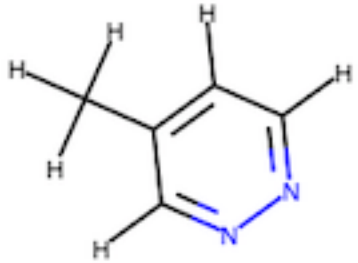
42	-4.27	-1.71	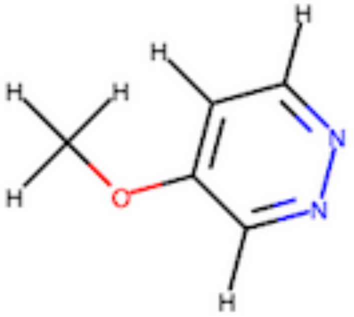
43	-4.20	-2.43	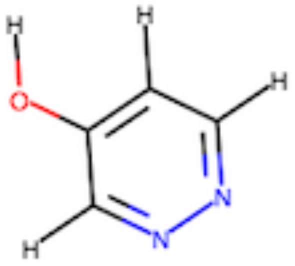
44	-5.70	-3.51	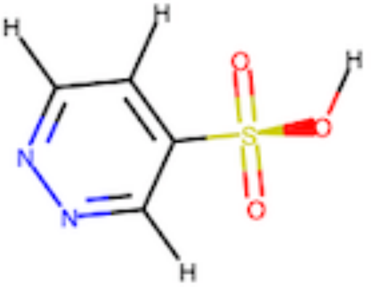

45	-4.10	-1.64	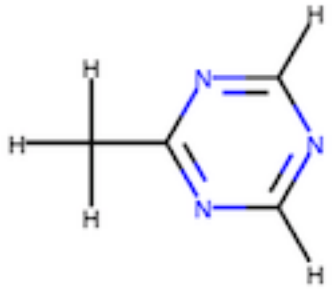
46	-3.40	-1.80	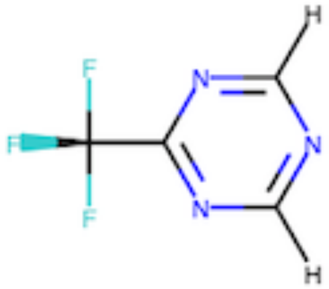
47	-3.91	-1.68	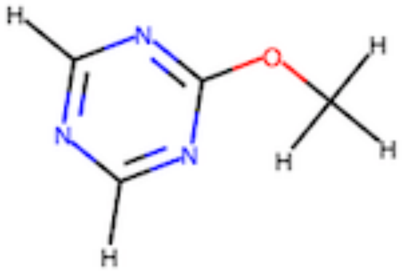
48	-5.23	-2.34	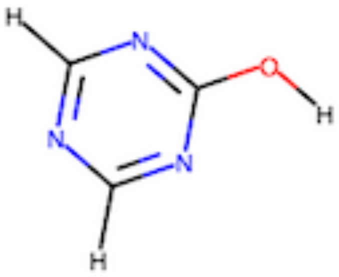
49	-6.26	-3.39	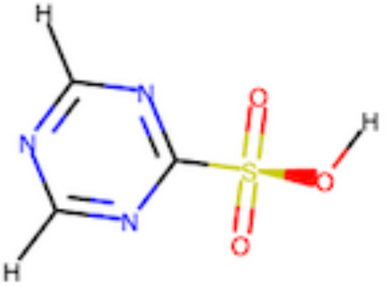

50	-4.15	-2.14	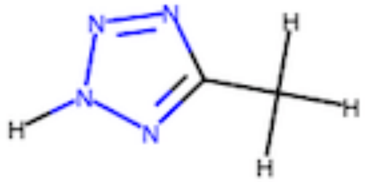
51	-3.94	-2.55	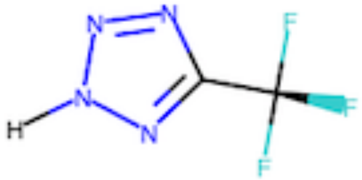
52	-4.15	-2.18	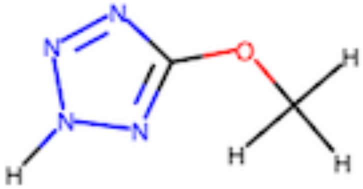
53	-5.44	-2.63	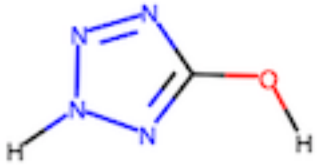
54	-3.87	-2.71	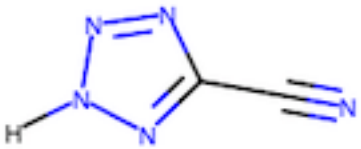

55	-6.82	-3.82	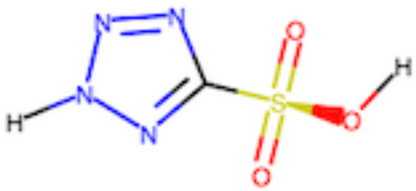
56	-5.34	-2.34	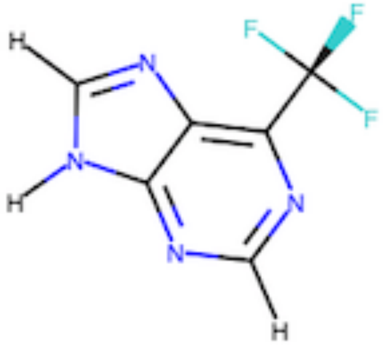
57	-5.65	-2.03	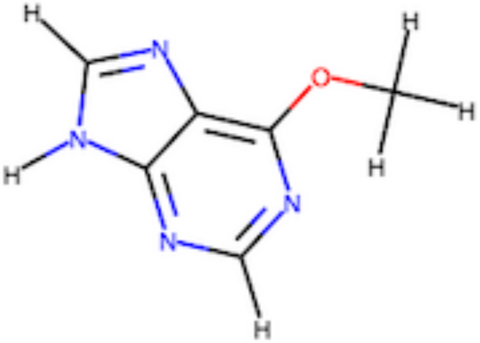
58	-5.62	-2.26	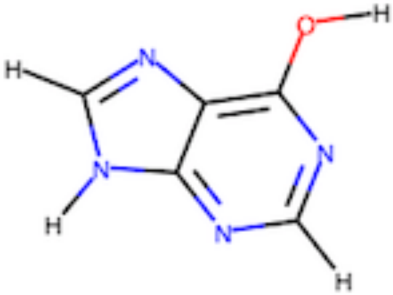

59	-5.30	-2.38	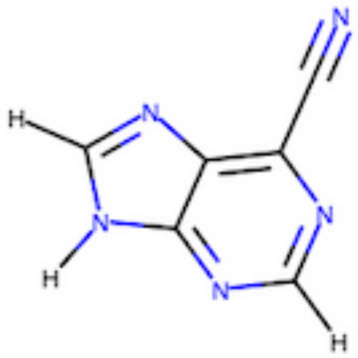
60	-5.41	-2.51	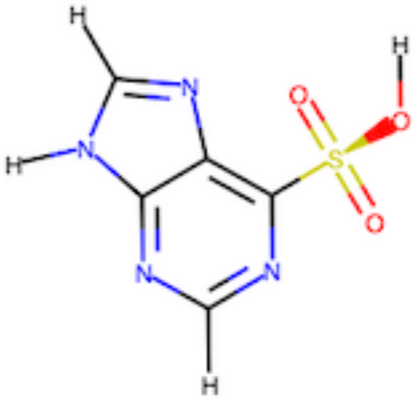
61	-5.53	-2.25	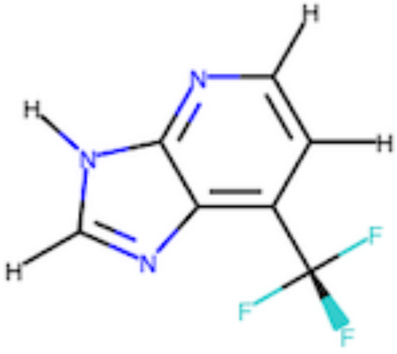
62	-6.02	-2.23	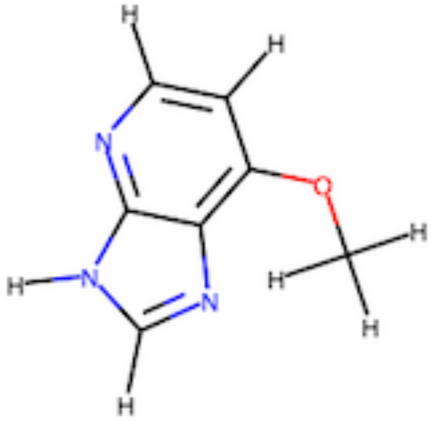

63	-6.06	-2.25	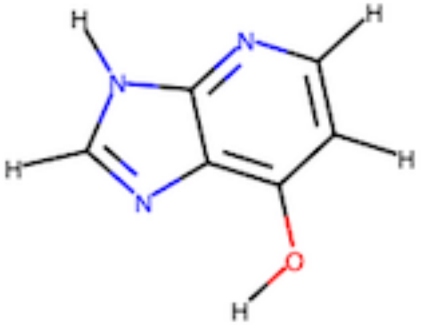
64	-5.46	-2.28	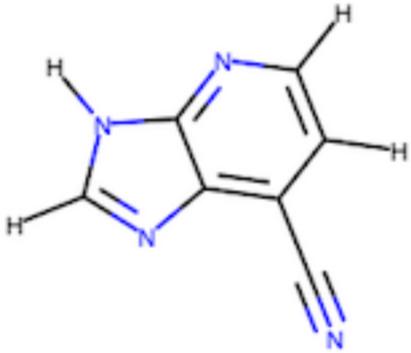
65	-5.56	-2.54	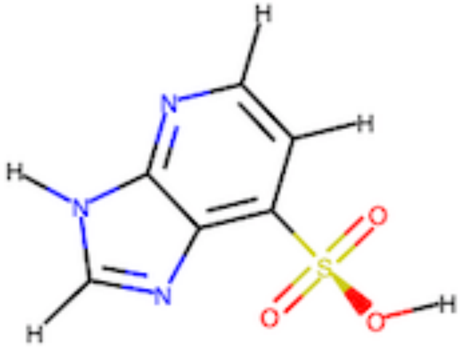

66	-3.14	-2.09	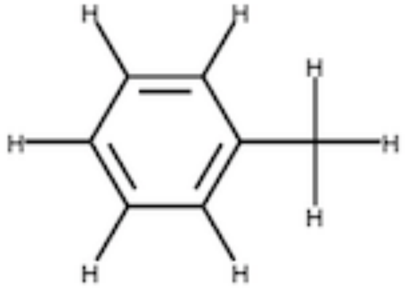
67	-2.88	-1.94	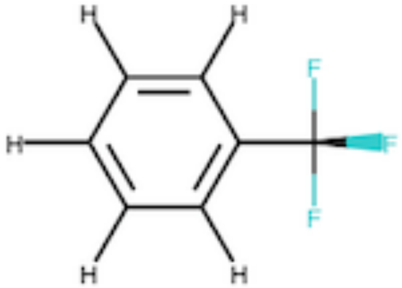
68	-3.20	-1.97	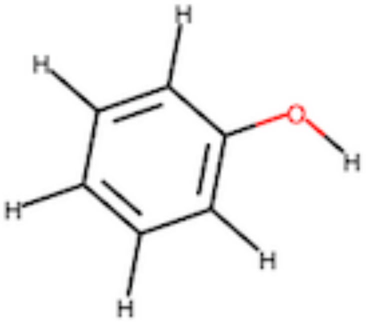

69	-3.42	-2.06	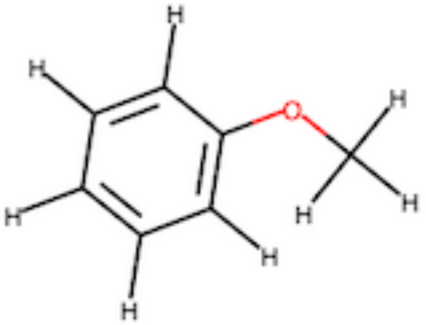
70	-5.52	-3.16	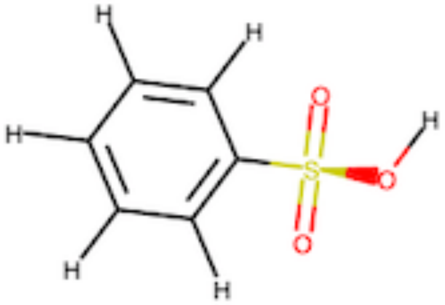
71	-4.81	-2.09	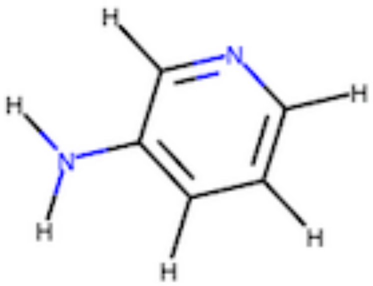
72	-5.34	-1.82	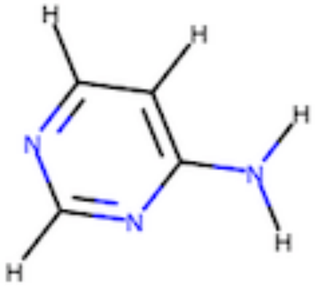

73	-4.43	-1.86	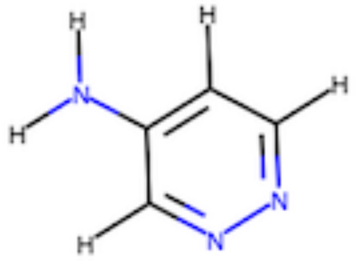
74	-5.22	-2.04	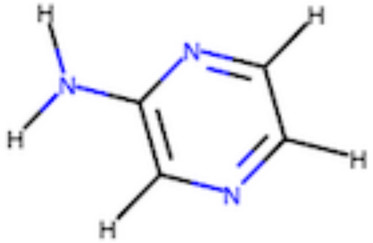
75	-5.20	-1.90	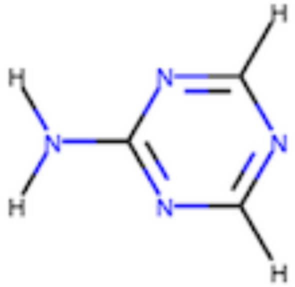
76	-4.72	-2.24	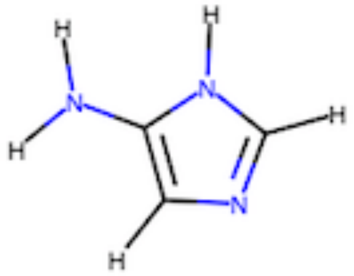
77	-4.71	-2.07	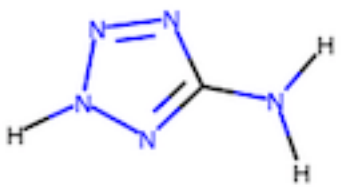

78	-5.79	-2.33	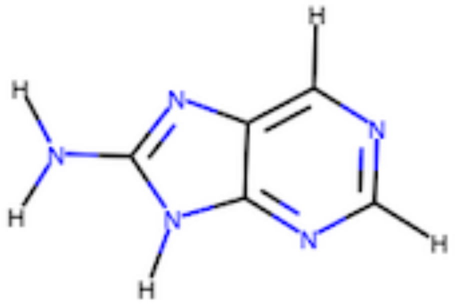
79	-5.89	-2.25	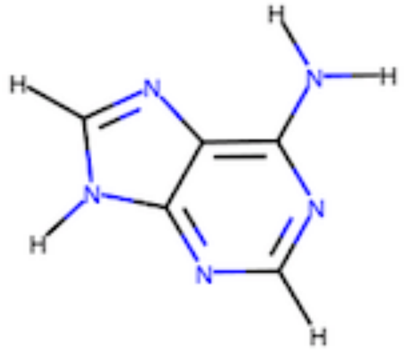
80	-6.35	-2.02	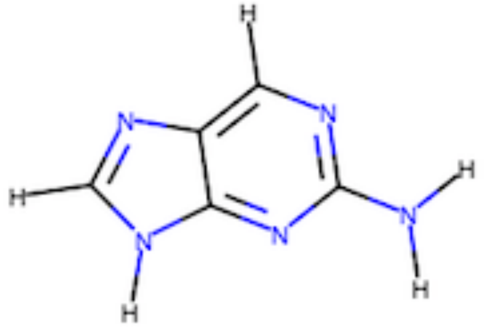
81	-6.13	-2.21	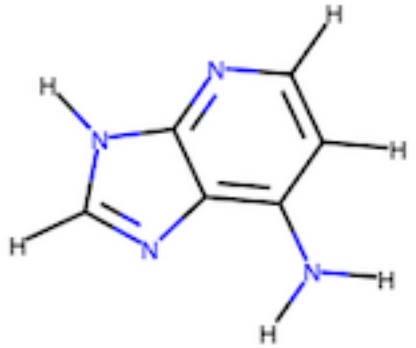

82	-6.04	-2.28	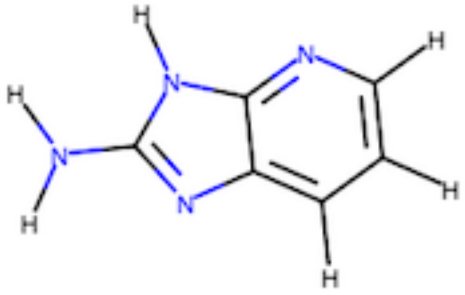
83	-3.96	-1.68	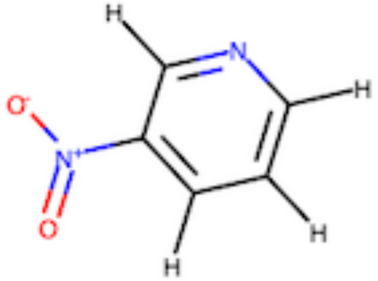
84	-4.04	-1.69	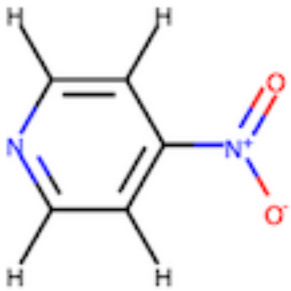
85	-3.55	-1.64	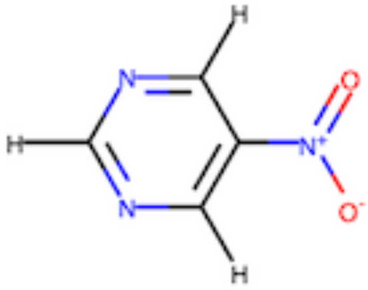

86	-3.57	-1.67	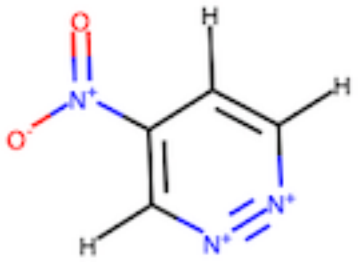
87	-3.94	-1.75	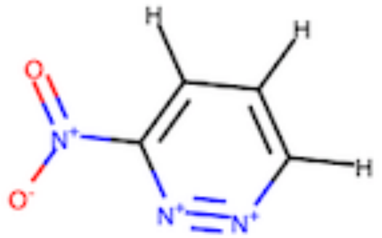
88	-3.60	-1.70	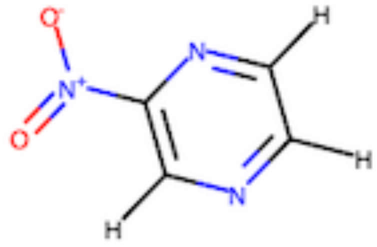
89	-3.41	-2.01	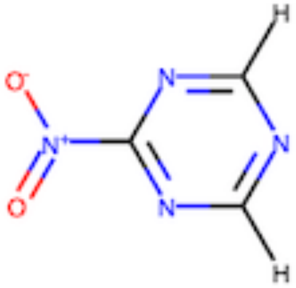

90	-4.54	-2.53	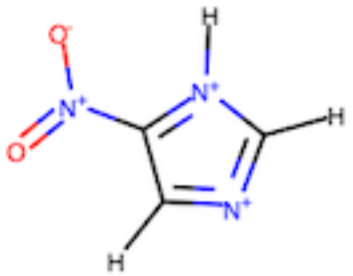
91	-4.48	-2.50	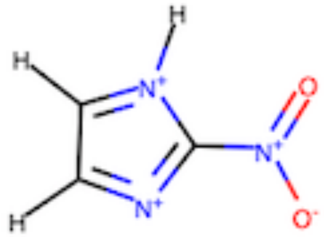
92	-3.98	-2.77	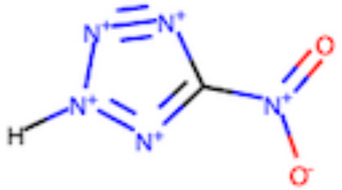
93	-5.29	-2.32	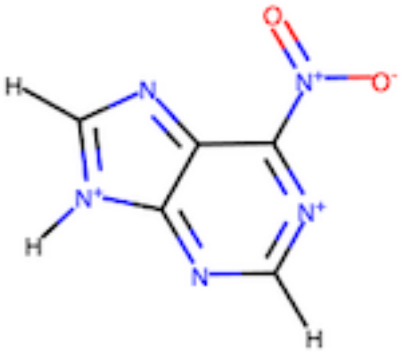

94	-5.02	-2.57	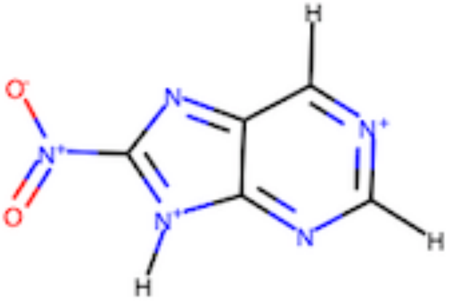
95	-5.43	-2.29	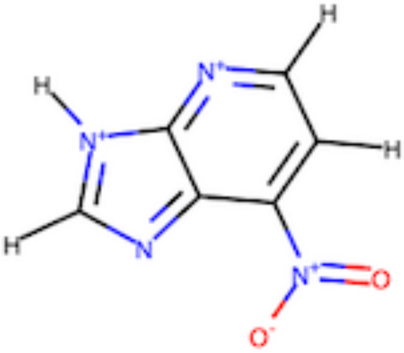
96	-5.18	-2.42	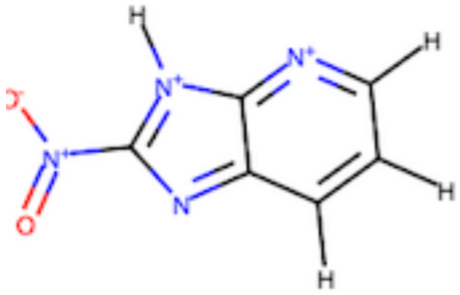
97	-3.19	-1.72	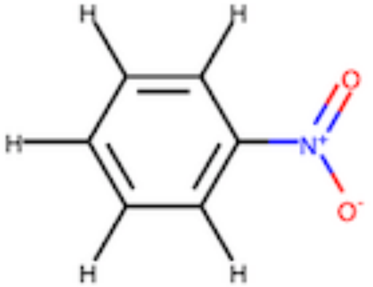

98	-4.58	-1.89	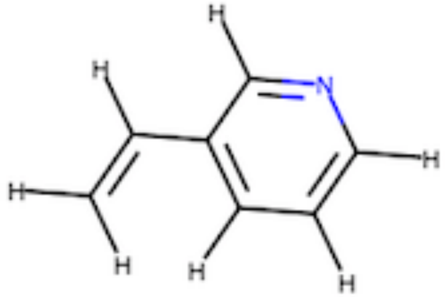
99	-4.23	-1.77	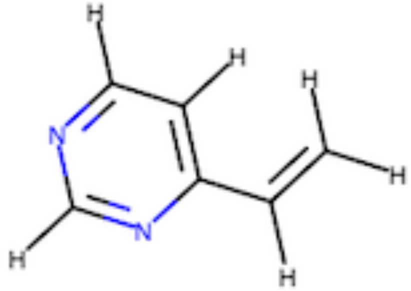
100	-4.08	-1.75	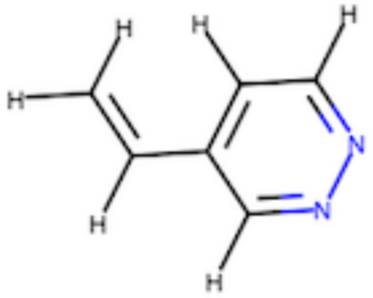

Supplementary Note 10. XYZ Coordinates and Energies of all DFT-optimized Structures

Supplementary Table 14. Molecules for the three iterations of active learning for PI, SOAP, CM, and BoB. The first iteration corresponds to molecules 1-40. The second set corresponds to molecules 41-80, and 81-120 corresponds to the third.

	PI	SOAP	CM	BoB
1	dsgdb9nsd_127513babel.xyz	dsgdb9nsd_027520babel.xyz	dsgdb9nsd_025924babel.xyz	dsgdb9nsd_026572babel.xyz
2	dsgdb9nsd_128165babel.xyz	dsgdb9nsd_027869babel.xyz	dsgdb9nsd_047090babel.xyz	dsgdb9nsd_029567babel.xyz
3	dsgdb9nsd_128158babel.xyz	dsgdb9nsd_133433babel.xyz	dsgdb9nsd_109208babel.xyz	dsgdb9nsd_029370babel.xyz
4	dsgdb9nsd_004514babel.xyz	dsgdb9nsd_027402babel.xyz	dsgdb9nsd_032461babel.xyz	dsgdb9nsd_027400babel.xyz
5	dsgdb9nsd_031100babel.xyz	dsgdb9nsd_024869babel.xyz	dsgdb9nsd_083107babel.xyz	dsgdb9nsd_027341babel.xyz
6	dsgdb9nsd_026710babel.xyz	dsgdb9nsd_131288babel.xyz	dsgdb9nsd_112329babel.xyz	dsgdb9nsd_026726babel.xyz
7	dsgdb9nsd_031557babel.xyz	dsgdb9nsd_132338babel.xyz	dsgdb9nsd_049792babel.xyz	dsgdb9nsd_027752babel.xyz
8	dsgdb9nsd_028534babel.xyz	dsgdb9nsd_131350babel.xyz	dsgdb9nsd_077363babel.xyz	dsgdb9nsd_026935babel.xyz
9	dsgdb9nsd_031149babel.xyz	dsgdb9nsd_130136babel.xyz	dsgdb9nsd_100178babel.xyz	dsgdb9nsd_026844babel.xyz
10	dsgdb9nsd_004525babel.xyz	dsgdb9nsd_132936babel.xyz	dsgdb9nsd_081360babel.xyz	dsgdb9nsd_026918babel.xyz
11	dsgdb9nsd_026709babel.xyz	dsgdb9nsd_131348babel.xyz	dsgdb9nsd_083506babel.xyz	dsgdb9nsd_029231babel.xyz
12	dsgdb9nsd_127511babel.xyz	dsgdb9nsd_132225babel.xyz	dsgdb9nsd_124213babel.xyz	dsgdb9nsd_029350babel.xyz
13	dsgdb9nsd_029791babel.xyz	dsgdb9nsd_132178babel.xyz	dsgdb9nsd_107612babel.xyz	dsgdb9nsd_027943babel.xyz
14	dsgdb9nsd_026705babel.xyz	dsgdb9nsd_133443babel.xyz	dsgdb9nsd_046959babel.xyz	dsgdb9nsd_075879babel.xyz
15	dsgdb9nsd_027908babel.xyz	dsgdb9nsd_132238babel.xyz	dsgdb9nsd_047586babel.xyz	dsgdb9nsd_064609babel.xyz
16	dsgdb9nsd_028542babel.xyz	dsgdb9nsd_132241babel.xyz	dsgdb9nsd_062488babel.xyz	dsgdb9nsd_027090babel.xyz
17	dsgdb9nsd_029783babel.xyz	dsgdb9nsd_133430babel.xyz	dsgdb9nsd_092864babel.xyz	dsgdb9nsd_027657babel.xyz
18	dsgdb9nsd_129638babel.xyz	dsgdb9nsd_131047babel.xyz	dsgdb9nsd_045815babel.xyz	dsgdb9nsd_081088babel.xyz
19	dsgdb9nsd_004513babel.xyz	dsgdb9nsd_132938babel.xyz	dsgdb9nsd_093391babel.xyz	dsgdb9nsd_027710babel.xyz
20	dsgdb9nsd_027911babel.xyz	dsgdb9nsd_131401babel.xyz	dsgdb9nsd_099251babel.xyz	dsgdb9nsd_027132babel.xyz
21	dsgdb9nsd_026708babel.xyz	dsgdb9nsd_004517babel.xyz	dsgdb9nsd_076893babel.xyz	dsgdb9nsd_028123babel.xyz
22	dsgdb9nsd_028531babel.xyz	dsgdb9nsd_003940babel.xyz	dsgdb9nsd_067548babel.xyz	dsgdb9nsd_075871babel.xyz
23	dsgdb9nsd_128129babel.xyz	dsgdb9nsd_027913babel.xyz	dsgdb9nsd_054720babel.xyz	dsgdb9nsd_028926babel.xyz
24	dsgdb9nsd_028529babel.xyz	dsgdb9nsd_027915babel.xyz	dsgdb9nsd_022569babel.xyz	dsgdb9nsd_029491babel.xyz
25	dsgdb9nsd_005091babel.xyz	dsgdb9nsd_003942babel.xyz	dsgdb9nsd_093309babel.xyz	dsgdb9nsd_067147babel.xyz
26	dsgdb9nsd_025935babel.xyz	dsgdb9nsd_024868babel.xyz	dsgdb9nsd_045581babel.xyz	dsgdb9nsd_029459babel.xyz
27	dsgdb9nsd_025890babel.xyz	dsgdb9nsd_129986babel.xyz	dsgdb9nsd_076541babel.xyz	dsgdb9nsd_064556babel.xyz
28	dsgdb9nsd_021542babel.xyz	dsgdb9nsd_132062babel.xyz	dsgdb9nsd_045427babel.xyz	dsgdb9nsd_027836babel.xyz
29	dsgdb9nsd_129636babel.xyz	dsgdb9nsd_131408babel.xyz	dsgdb9nsd_076539babel.xyz	dsgdb9nsd_029163babel.xyz
30	dsgdb9nsd_004517babel.xyz	dsgdb9nsd_027515babel.xyz	dsgdb9nsd_082653babel.xyz	dsgdb9nsd_076218babel.xyz
31	dsgdb9nsd_029704babel.xyz	dsgdb9nsd_130054babel.xyz	dsgdb9nsd_076784babel.xyz	dsgdb9nsd_098949babel.xyz
32	dsgdb9nsd_029714babel.xyz	dsgdb9nsd_003937babel.xyz	dsgdb9nsd_112216babel.xyz	dsgdb9nsd_026613babel.xyz
33	dsgdb9nsd_003937babel.xyz	dsgdb9nsd_004244babel.xyz	dsgdb9nsd_112352babel.xyz	dsgdb9nsd_053993babel.xyz

34	dsgdb9nsd_028536babel.xyz	dsgdb9nsd_132935babel.xyz	dsgdb9nsd_127751babel.xyz	dsgdb9nsd_027917babel.xyz
35	dsgdb9nsd_129637babel.xyz	dsgdb9nsd_132233babel.xyz	dsgdb9nsd_045877babel.xyz	dsgdb9nsd_057173babel.xyz
36	dsgdb9nsd_028535babel.xyz	dsgdb9nsd_129502babel.xyz	dsgdb9nsd_053818babel.xyz	dsgdb9nsd_028927babel.xyz
37	dsgdb9nsd_129639babel.xyz	dsgdb9nsd_022906babel.xyz	dsgdb9nsd_046040babel.xyz	dsgdb9nsd_075737babel.xyz
38	dsgdb9nsd_021543babel.xyz	dsgdb9nsd_004532babel.xyz	dsgdb9nsd_116436babel.xyz	dsgdb9nsd_070169babel.xyz
39	dsgdb9nsd_131257babel.xyz	dsgdb9nsd_025890babel.xyz	dsgdb9nsd_080842babel.xyz	dsgdb9nsd_077806babel.xyz
40	dsgdb9nsd_026701babel.xyz	dsgdb9nsd_133431babel.xyz	dsgdb9nsd_081933babel.xyz	dsgdb9nsd_029326babel.xyz
41	dsgdb9nsd_027936babel.xyz	dsgdb9nsd_133426babel.xyz	dsgdb9nsd_028135babel.xyz	dsgdb9nsd_028837babel.xyz
42	dsgdb9nsd_003940babel.xyz	dsgdb9nsd_027936babel.xyz	dsgdb9nsd_028185babel.xyz	dsgdb9nsd_028125babel.xyz
43	dsgdb9nsd_003882babel.xyz	dsgdb9nsd_000860babel.xyz	dsgdb9nsd_027081babel.xyz	dsgdb9nsd_027202babel.xyz
44	dsgdb9nsd_004512babel.xyz	dsgdb9nsd_129389babel.xyz	dsgdb9nsd_067368babel.xyz	dsgdb9nsd_024471babel.xyz
45	dsgdb9nsd_031067babel.xyz	dsgdb9nsd_132800babel.xyz	dsgdb9nsd_029616babel.xyz	dsgdb9nsd_029461babel.xyz
46	dsgdb9nsd_027942babel.xyz	dsgdb9nsd_027908babel.xyz	dsgdb9nsd_028394babel.xyz	dsgdb9nsd_046168babel.xyz
47	dsgdb9nsd_004511babel.xyz	dsgdb9nsd_131359babel.xyz	dsgdb9nsd_131537babel.xyz	dsgdb9nsd_028178babel.xyz
48	dsgdb9nsd_004518babel.xyz	dsgdb9nsd_129223babel.xyz	dsgdb9nsd_022018babel.xyz	dsgdb9nsd_027432babel.xyz
49	dsgdb9nsd_004530babel.xyz	dsgdb9nsd_132628babel.xyz	dsgdb9nsd_029099babel.xyz	dsgdb9nsd_028986babel.xyz
50	dsgdb9nsd_021045babel.xyz	dsgdb9nsd_027653babel.xyz	dsgdb9nsd_131561babel.xyz	dsgdb9nsd_028836babel.xyz
51	dsgdb9nsd_031114babel.xyz	dsgdb9nsd_132612babel.xyz	dsgdb9nsd_080165babel.xyz	dsgdb9nsd_029492babel.xyz
52	dsgdb9nsd_129384babel.xyz	dsgdb9nsd_028534babel.xyz	dsgdb9nsd_131410babel.xyz	dsgdb9nsd_028980babel.xyz
53	dsgdb9nsd_030849babel.xyz	dsgdb9nsd_131273babel.xyz	dsgdb9nsd_066893babel.xyz	dsgdb9nsd_043555babel.xyz
54	dsgdb9nsd_030245babel.xyz	dsgdb9nsd_004085babel.xyz	dsgdb9nsd_028193babel.xyz	dsgdb9nsd_129855babel.xyz
55	dsgdb9nsd_131168babel.xyz	dsgdb9nsd_130160babel.xyz	dsgdb9nsd_029539babel.xyz	dsgdb9nsd_025222babel.xyz
56	dsgdb9nsd_031052babel.xyz	dsgdb9nsd_027911babel.xyz	dsgdb9nsd_131552babel.xyz	dsgdb9nsd_129912babel.xyz
57	dsgdb9nsd_031084babel.xyz	dsgdb9nsd_131343babel.xyz	dsgdb9nsd_131557babel.xyz	dsgdb9nsd_027441babel.xyz
58	dsgdb9nsd_025925babel.xyz	dsgdb9nsd_129388babel.xyz	dsgdb9nsd_027917babel.xyz	dsgdb9nsd_027221babel.xyz
59	dsgdb9nsd_021055babel.xyz	dsgdb9nsd_000880babel.xyz	dsgdb9nsd_029160babel.xyz	dsgdb9nsd_028984babel.xyz
60	dsgdb9nsd_004515babel.xyz	dsgdb9nsd_004514babel.xyz	dsgdb9nsd_131533babel.xyz	dsgdb9nsd_029060babel.xyz
61	dsgdb9nsd_003873babel.xyz	dsgdb9nsd_132642babel.xyz	dsgdb9nsd_123593babel.xyz	dsgdb9nsd_028987babel.xyz
62	dsgdb9nsd_030860babel.xyz	dsgdb9nsd_131357babel.xyz	dsgdb9nsd_027089babel.xyz	dsgdb9nsd_029051babel.xyz
63	dsgdb9nsd_029159babel.xyz	dsgdb9nsd_131371babel.xyz	dsgdb9nsd_081525babel.xyz	dsgdb9nsd_043603babel.xyz
64	dsgdb9nsd_031057babel.xyz	dsgdb9nsd_027912babel.xyz	dsgdb9nsd_128700babel.xyz	dsgdb9nsd_028832babel.xyz
65	dsgdb9nsd_005116babel.xyz	dsgdb9nsd_003882babel.xyz	dsgdb9nsd_029104babel.xyz	dsgdb9nsd_028299babel.xyz
66	dsgdb9nsd_004942babel.xyz	dsgdb9nsd_028521babel.xyz	dsgdb9nsd_029057babel.xyz	dsgdb9nsd_129861babel.xyz
67	dsgdb9nsd_131262babel.xyz	dsgdb9nsd_132379babel.xyz	dsgdb9nsd_047108babel.xyz	dsgdb9nsd_080207babel.xyz
68	dsgdb9nsd_031061babel.xyz	dsgdb9nsd_132607babel.xyz	dsgdb9nsd_054393babel.xyz	dsgdb9nsd_028656babel.xyz
69	dsgdb9nsd_030891babel.xyz	dsgdb9nsd_129236babel.xyz	dsgdb9nsd_032311babel.xyz	dsgdb9nsd_025841babel.xyz
70	dsgdb9nsd_027654babel.xyz	dsgdb9nsd_027918babel.xyz	dsgdb9nsd_132627babel.xyz	dsgdb9nsd_129916babel.xyz
71	dsgdb9nsd_031005babel.xyz	dsgdb9nsd_027914babel.xyz	dsgdb9nsd_132010babel.xyz	dsgdb9nsd_029053babel.xyz
72	dsgdb9nsd_031101babel.xyz	dsgdb9nsd_132652babel.xyz	dsgdb9nsd_028896babel.xyz	dsgdb9nsd_043428babel.xyz

73	dsgdb9nsd_030861babel.xyz	dsgdb9nsd_022030babel.xyz	dsgdb9nsd_029470babel.xyz	dsgdb9nsd_024535babel.xyz
74	dsgdb9nsd_004532babel.xyz	dsgdb9nsd_028546babel.xyz	dsgdb9nsd_045918babel.xyz	dsgdb9nsd_024534babel.xyz
75	dsgdb9nsd_031063babel.xyz	dsgdb9nsd_028535babel.xyz	dsgdb9nsd_028254babel.xyz	dsgdb9nsd_027443babel.xyz
76	dsgdb9nsd_030850babel.xyz	dsgdb9nsd_028533babel.xyz	dsgdb9nsd_045949babel.xyz	dsgdb9nsd_029048babel.xyz
77	dsgdb9nsd_021210babel.xyz	dsgdb9nsd_026710babel.xyz	dsgdb9nsd_130326babel.xyz	dsgdb9nsd_130170babel.xyz
78	dsgdb9nsd_030906babel.xyz	dsgdb9nsd_129222babel.xyz	dsgdb9nsd_023299babel.xyz	dsgdb9nsd_024529babel.xyz
79	dsgdb9nsd_028547babel.xyz	dsgdb9nsd_132390babel.xyz	dsgdb9nsd_098142babel.xyz	dsgdb9nsd_024497babel.xyz
80	dsgdb9nsd_131334babel.xyz	dsgdb9nsd_132802babel.xyz	dsgdb9nsd_127750babel.xyz	dsgdb9nsd_024498babel.xyz
81	dsgdb9nsd_020994babel.xyz	dsgdb9nsd_132656babel.xyz	dsgdb9nsd_130318babel.xyz	dsgdb9nsd_024531babel.xyz
82	dsgdb9nsd_004465babel.xyz	dsgdb9nsd_029433babel.xyz	dsgdb9nsd_130616babel.xyz	dsgdb9nsd_024500babel.xyz
83	dsgdb9nsd_004482babel.xyz	dsgdb9nsd_000854babel.xyz	dsgdb9nsd_026432babel.xyz	dsgdb9nsd_024504babel.xyz
84	dsgdb9nsd_031645babel.xyz	dsgdb9nsd_027745babel.xyz	dsgdb9nsd_129794babel.xyz	dsgdb9nsd_024527babel.xyz
85	dsgdb9nsd_126708babel.xyz	dsgdb9nsd_004294babel.xyz	dsgdb9nsd_130491babel.xyz	dsgdb9nsd_028115babel.xyz
86	dsgdb9nsd_004553babel.xyz	dsgdb9nsd_026843babel.xyz	dsgdb9nsd_030380babel.xyz	dsgdb9nsd_024472babel.xyz
87	dsgdb9nsd_126772babel.xyz	dsgdb9nsd_004669babel.xyz	dsgdb9nsd_029957babel.xyz	dsgdb9nsd_030226babel.xyz
88	dsgdb9nsd_030791babel.xyz	dsgdb9nsd_027753babel.xyz	dsgdb9nsd_127713babel.xyz	dsgdb9nsd_030247babel.xyz
89	dsgdb9nsd_004547babel.xyz	dsgdb9nsd_004530babel.xyz	dsgdb9nsd_129974babel.xyz	dsgdb9nsd_027766babel.xyz
90	dsgdb9nsd_031502babel.xyz	dsgdb9nsd_132457babel.xyz	dsgdb9nsd_049599babel.xyz	dsgdb9nsd_030259babel.xyz
91	dsgdb9nsd_005113babel.xyz	dsgdb9nsd_132632babel.xyz	dsgdb9nsd_075292babel.xyz	dsgdb9nsd_028334babel.xyz
92	dsgdb9nsd_031095babel.xyz	dsgdb9nsd_129489babel.xyz	dsgdb9nsd_133390babel.xyz	dsgdb9nsd_024324babel.xyz
93	dsgdb9nsd_022249babel.xyz	dsgdb9nsd_004381babel.xyz	dsgdb9nsd_128118babel.xyz	dsgdb9nsd_028504babel.xyz
94	dsgdb9nsd_031776babel.xyz	dsgdb9nsd_028197babel.xyz	dsgdb9nsd_133235babel.xyz	dsgdb9nsd_024495babel.xyz
95	dsgdb9nsd_004478babel.xyz	dsgdb9nsd_132312babel.xyz	dsgdb9nsd_075126babel.xyz	dsgdb9nsd_105980babel.xyz
96	dsgdb9nsd_026329babel.xyz	dsgdb9nsd_132673babel.xyz	dsgdb9nsd_039609babel.xyz	dsgdb9nsd_031875babel.xyz
97	dsgdb9nsd_126782babel.xyz	dsgdb9nsd_004717babel.xyz	dsgdb9nsd_025111babel.xyz	dsgdb9nsd_029747babel.xyz
98	dsgdb9nsd_030858babel.xyz	dsgdb9nsd_028089babel.xyz	dsgdb9nsd_132113babel.xyz	dsgdb9nsd_029815babel.xyz
99	dsgdb9nsd_029832babel.xyz	dsgdb9nsd_027387babel.xyz	dsgdb9nsd_127525babel.xyz	dsgdb9nsd_024470babel.xyz
100	dsgdb9nsd_003871babel.xyz	dsgdb9nsd_027707babel.xyz	dsgdb9nsd_029874babel.xyz	dsgdb9nsd_024502babel.xyz
101	dsgdb9nsd_033118babel.xyz	dsgdb9nsd_028754babel.xyz	dsgdb9nsd_040543babel.xyz	dsgdb9nsd_029829babel.xyz
102	dsgdb9nsd_030228babel.xyz	dsgdb9nsd_132386babel.xyz	dsgdb9nsd_050304babel.xyz	dsgdb9nsd_024530babel.xyz
103	dsgdb9nsd_004468babel.xyz	dsgdb9nsd_028001babel.xyz	dsgdb9nsd_028140babel.xyz	dsgdb9nsd_024536babel.xyz
104	dsgdb9nsd_004920babel.xyz	dsgdb9nsd_129524babel.xyz	dsgdb9nsd_024262babel.xyz	dsgdb9nsd_024501babel.xyz
105	dsgdb9nsd_003877babel.xyz	dsgdb9nsd_027654babel.xyz	dsgdb9nsd_045018babel.xyz	dsgdb9nsd_028798babel.xyz
106	dsgdb9nsd_030894babel.xyz	dsgdb9nsd_000857babel.xyz	dsgdb9nsd_029935babel.xyz	dsgdb9nsd_028483babel.xyz
107	dsgdb9nsd_030880babel.xyz	dsgdb9nsd_021881babel.xyz	dsgdb9nsd_130207babel.xyz	dsgdb9nsd_024468babel.xyz
108	dsgdb9nsd_030892babel.xyz	dsgdb9nsd_025826babel.xyz	dsgdb9nsd_025223babel.xyz	dsgdb9nsd_029935babel.xyz
109	dsgdb9nsd_030843babel.xyz	dsgdb9nsd_004279babel.xyz	dsgdb9nsd_028186babel.xyz	dsgdb9nsd_032177babel.xyz
110	dsgdb9nsd_031017babel.xyz	dsgdb9nsd_025791babel.xyz	dsgdb9nsd_082158babel.xyz	dsgdb9nsd_032278babel.xyz
111	dsgdb9nsd_031040babel.xyz	dsgdb9nsd_028759babel.xyz	dsgdb9nsd_094427babel.xyz	dsgdb9nsd_032279babel.xyz

112	dsgdb9nsd_027725babel.xyz	dsgdb9nsd_021887babel.xyz	dsgdb9nsd_127524babel.xyz	dsgdb9nsd_026733babel.xyz
113	dsgdb9nsd_005110babel.xyz	dsgdb9nsd_004749babel.xyz	dsgdb9nsd_027116babel.xyz	dsgdb9nsd_029823babel.xyz
114	dsgdb9nsd_027730babel.xyz	dsgdb9nsd_027917babel.xyz	dsgdb9nsd_131418babel.xyz	dsgdb9nsd_032281babel.xyz
115	dsgdb9nsd_131278babel.xyz	dsgdb9nsd_026810babel.xyz	dsgdb9nsd_127511babel.xyz	dsgdb9nsd_028170babel.xyz
116	dsgdb9nsd_031089babel.xyz	dsgdb9nsd_132391babel.xyz	dsgdb9nsd_030004babel.xyz	dsgdb9nsd_026757babel.xyz
117	dsgdb9nsd_004459babel.xyz	dsgdb9nsd_132305babel.xyz	dsgdb9nsd_030370babel.xyz	dsgdb9nsd_027987babel.xyz
118	dsgdb9nsd_022592babel.xyz	dsgdb9nsd_026804babel.xyz	dsgdb9nsd_029944babel.xyz	dsgdb9nsd_032172babel.xyz
119	dsgdb9nsd_000880babel.xyz	dsgdb9nsd_027393babel.xyz	dsgdb9nsd_024201babel.xyz	dsgdb9nsd_029874babel.xyz
120	dsgdb9nsd_027671babel.xyz	dsgdb9nsd_026840babel.xyz	dsgdb9nsd_133257babel.xyz	dsgdb9nsd_027292babel.xyz

Supplementary References

(1) Maroon, C. R.; Townsend, J.; Gmernicki, K. R.; Harrigan, D. J.; Sundell, B. J.; Lawrence, J. A.; Mahurin, S. M.; Vogiatzis, K. D.; Long, B. K. Elimination of CO 2 /N 2 Langmuir Sorption and Promotion of "n 2 -Phobicity" within High- T g Glassy Membranes. *Macromolecules* **2019**, *52* (4), 1589–1600.

(2) Jorgensen, W. L.; Maxwell, D. S.; Tirado-Rives, J. Development and Testing of the OPLS All-Atom Force Field on Conformational Energetics and Properties of Organic Liquids. *J. Am. Chem. Soc.* **1996**, *118* (45), 11225–11236.

(3) Dodda, L. S.; De Vaca, I. C.; Tirado-Rives, J.; Jorgensen, W. L. LigParGen Web Server: An Automatic OPLS-AA Parameter Generator for Organic Ligands. *Nucleic Acids Res.* **2017**, *45* (W1), W331–W336.

(4) Plimpton, S. Fast Parallel Algorithms for Short-Range Molecular Dynamics. *J. Comput. Phys.* **1995**, *117* (1), 1–19.

(5) Furche, F.; Ahlrichs, R.; Hättig, C.; Klopper, W.; Sierka, M.; Weigend, F. Turbomole. *Wiley Interdiscip. Rev. Comput. Mol. Sci.* **2014**, *4* (2), 91–100.

(6) Adamo, C.; Barone, V. Toward Reliable Density Functional Methods without Adjustable Parameters: The PBE0 Model. *J. Chem. Phys.* **1999**, *110* (13), 6158–6170.

(7) Weigend, F.; Ahlrichs, R. Balanced Basis Sets of Split Valence, Triple Zeta Valence and Quadruple Zeta Valence Quality for H to Rn: Design and Assessment of Accuracy. *Phys. Chem. Chem. Phys.* **2005**, *7* (18), 3297–3305.

(8) Grimme, S.; Antony, J.; Ehrlich, S.; Krieg, H. A Consistent and Accurate Ab Initio Parametrization of Density Functional Dispersion Correction (DFT-D) for the 94 Elements H-Pu. *J. Chem. Phys.* **2010**, *132* (15), 154104.

(9) Grimme, S.; Ehrlich, S.; Goerigk, L. Effect of the Damping Function in Dispersion Corrected Density Functional Theory. *J. Comput. Chem.* **2011**, *32* (7), 1456–1465.

(10) Mavrandonakis, A.; Vogiatzis, K. D.; Boese, A. D.; Fink, K.; Heine, T.; Klopper, W. Ab Initio Study of the Adsorption of Small Molecules on Metal-Organic Frameworks with Oxo-Centered Trimetallic Building Units: The Role of the Undercoordinated Metal Ion. *Inorg. Chem.* **2015**, *54* (17), 8251–8263.

(11) Vogiatzis, K. D.; Klopper, W.; Friedrich, J. Non-Covalent Interactions of CO<inf>2</Inf> with Functional Groups of Metal-Organic Frameworks from a CCSD(T) Scheme Applicable to Large Systems. *J. Chem. Theory Comput.* **2015**, *11* (4), 1574–1584.

(12) Hättig, C. Optimization of Auxiliary Basis Sets for RI-MP2 and RI-CC2 Calculations: Core–Valence and Quintuple-ζ Basis Sets for H to Ar and QZVPP Basis Sets for Li to Kr. *Phys. Chem. Chem. Phys.* **2005**, *7* (1), 59–66.

(13) Bauer, U. Ripser: Efficient Computation of Vietoris-Rips Persistence Barcodes. **2019**, 1–16.

(14) Saul, N. Persim. https://pypi.org/project/persim/.

(15) Jin, Z.; Du, X.; Xu, Y.; Deng, Y.; Liu, M.; Zhao, Y.; Zhang, B.; Li, X.; Zhang, L.; Peng, C.; et al. Structure of Mpro from COVID-19 Virus and Discovery of Its Inhibitors. *bioRxiv* **2020**, https://doi.org/10.1101/2020.02.26.964882.

(16) Pedregosa, F.; Michel, V.; Grisel, O.; Blondel, M.; Prettenhofer, P.; Weiss, R.; Vanderplas, J.; Cournapeau, D.; Varoquaux, G.; Gramfort, A.; et al. Scikit-Learn: Machine Learning in Python. *J. Mach. Learn. Res.* **2011**, *12*, 2825–2830.

(17) Bartók, A. P.; Kondor, R.; Csányi, G. On Representing Chemical Environments. *Phys. Rev. B - Condens. Matter Mater. Phys.* **2013**, *87* (18), 1–16.

(18) Himanen, L.; Jäger, M. O. J.; Morooka, E. V.; Federici Canova, F.; Ranawat, Y. S.; Gao, D. Z.; Rinke, P.; Foster, A. S. DScribe: Library of Descriptors for Machine Learning in Materials Science. *Comput. Phys. Commun.* **2020**, *247*, 106949.
(19) De, S.; Bartók, A. P.; Csányi, G.; Ceriotti, M. Comparing Molecules and Solids across Structural and Alchemical Space. *Phys. Chem. Chem. Phys.* **2016**, *18* (20), 13754–13769.
(20) Hansen, K.; Biegler, F.; Ramakrishnan, R.; Pronobis, W.; Von Lilienfeld, O. A.; Müller, K. R.; Tkatchenko, A. Machine Learning Predictions of Molecular Properties: Accurate Many-Body Potentials and Nonlocality in Chemical Space. *J. Phys. Chem. Lett.* **2015**, *6* (12), 2326–2331.
(21) Rupp, M.; Tkatchenko, A.; Müller, K.-R.; Anatole Von Lilienfeld, O. Fast and Accurate Modeling of Molecular Atomization Energies with Machine Learning. *Phys. Rev. Lett.* **2012**, *108* (5), 058301.
(22) Christensen, A. S.; Faber, F. A.; Von Lilienfeld, O. A. Operators in Quantum Machine Learning: Response Properties in Chemical Space. *J. Chem. Phys.* **2019**, *150* (6), 1–15.
(23) Christensen, A.; Faber, F. A.; Bratholm, L.; Tkatchenko, A.; Muller, K.; Von Lilienfeld, O. A. Qml: A python toolkit for quantum machine learning.
